# Oral amelanotic malignant melanoma: a case report

**DOI:** 10.11604/pamj.2020.37.350.27330

**Published:** 2020-12-16

**Authors:** Zakaria Aziz, Salma Aboulouidad, Mohamed El Bouihi, Nadia Mansouri Hattab, Mohamed Chehbouni, Abdelaziz Raji

**Affiliations:** 1 Maxillofacial Surgery Department, University Hospital Center Mohammed VI, Marrakesh, Morocco,; 2 Department of Otorhinolaryngology, University Hospital Center Mohammed VI, Marrakesh, Morocco

**Keywords:** Malignant melanoma, amelanotic, oral cavity, maxillary gingiva, case report

## Abstract

Amelanotic malignant melanoma is an extremely rare and aggressive oral tumor. Herein we report the case of a 42-year-old woman presented with a painful growth in anterior maxillary region. Intra-oral examination showed a non-pigmented exophytic mass occupying the anterior maxillary sector. Incisional biopsy with immunohistochemistry examination revealed a malignant melanoma as it strongly expressed melan A and S-100. Facial computed tomography showed extension to the maxillary bone and hard palate. After thoraco-abdominal computed tomography revealing absence of metastasis, tumor resection was performed respecting 2cm security margin. Oral localization of malignant melanoma is rare especially its amelanotic variant. Lack of pigmentation makes the diagnosis more difficult, usually resulting in treatment delay and making the prognosis even worse. Early detection by histological and immunochemistry examination combined to wide resection are the keys to improving the survival for patients with oral amelanotic melanoma.

## Introduction

Malignant melanomas arising in the oral cavity are extremely rare neoplasms accounting for less than 1% of all melanomas and for 0.5% of all oral malignancies [1]. It´s an adulthood cancer, rarely identified under the age of 20 years with a male predilection, and the most frequently affected sites are the hard palate and the gingiva [2,3]. The poor prognosis of this cancer is known with an overall 5 years survival rate at 15% [2]. In rare instances (2 to 8%), melanin may be missing and the lesion is referred as amelanotic malignant melanoma (AMM) [4]. Oral amelanotic malignant melanoma presents a unique diagnostic challenge since pigmentation, the main clinical feature of melanoma, is missing. This usually results in treatment delay and makes the prognosis even poorer [4,5].

We report here clinic-pathological findings and treatment strategy of a unique case of an oral amelanotic melanoma located at the maxillary gingiva. We highlight the usefulness of immunohistochemistry for the correct and early diagnosis of this cancer.

## Patient and observation

On December 2019, a 42-year-old woman was referred by the otolaryngology department to the oral and maxillofacial surgery department of Marrakesh University Hospital, with a three months history of a progressively enlarging painful growth in anterior maxillary region. It was stated that the lesion was initially a non-painful gingival swelling that recently increased in size with the onset of pain. The patient reported the loss of the left central incisor and recently noticed bleeding during meals. The patient had no notable medical history.

**Figure 1:**
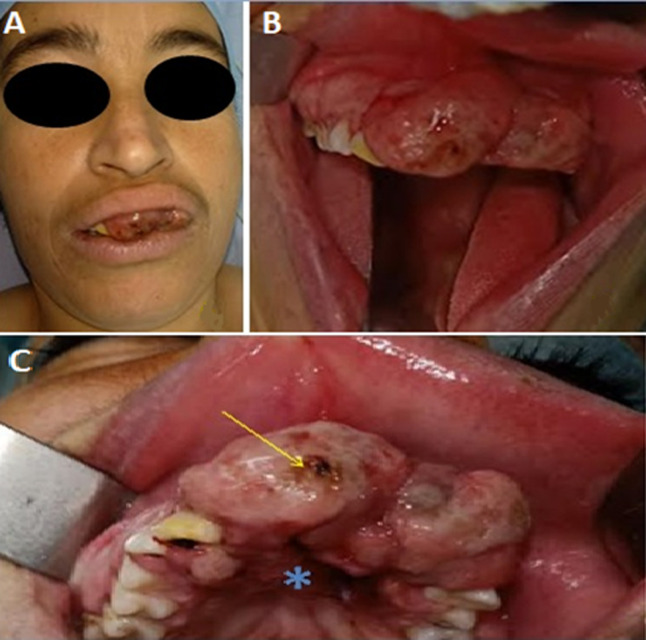
A) extraoral view showing an exophytic mass causing mild labial superior swelling and preventing lips from closing; B) intraoral view of a pink non-pigmented 4cm sized tumor of the maxillary gingival; C) intraoral view demonstrating blood spots (arrow) and invasion of hard palate (*)

Extra-oral examination demonstrated an exophytic mass causing mild labial superior swelling and preventing lips from closing. No apparent lymph node enlargement was found. Intra oral examination showed a pink, sessile, non-pigmented mass measuring 4 cm. The tumor was firm with an uneven surface and some residual blood spots. It occupied the anterior maxillary sector and the corresponding vestibule area. It was extending to the anterior third of hard palate and pushing apart teeth 11 and 22 which were highly mobile (Figure 1).

**Figure 2:**
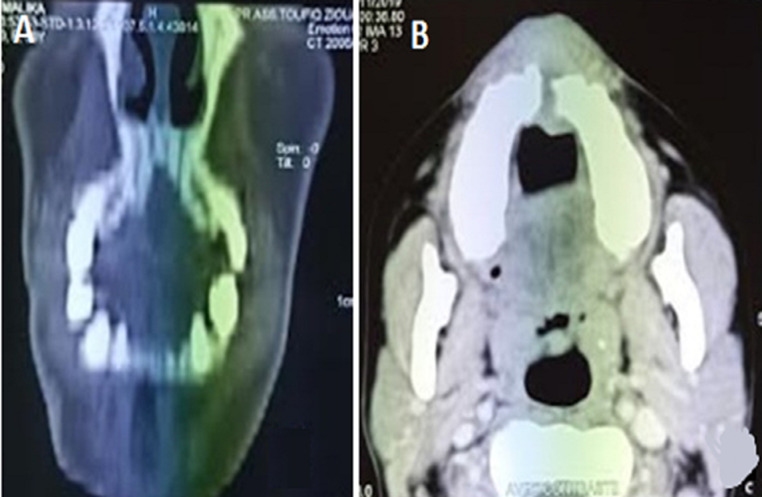
A) CT coronal view showing palatal bone destruction; B) CT axial view demonstrating soft tissue mass arising in the anterior maxilla with bone erosion

Craniofacial computed tomography showed a superior soft tissue process extending to the maxillary bone and hard palate with integrity of maxillary sinus (Figure 2). A carcinoma was suspected and an incisional biopsy was performed. Histological examination showed a poorly differentiated proliferation made up of spindle-shaped cells with round or oval nuclei moderately increased in size; hyperchromatic with numerous mitotic figures. No melanin pigment was identified in the cells. Immunohistochemistry was required to conclude to the diagnosis of malignant melanoma as the tumor cells strongly expressed melan A and S-100 whereas AE1/AE3, CD34, P63 were negative. There was no evidence of distant metastasis on the thoraco-abdominal computed tomography.

**Figure 3:**
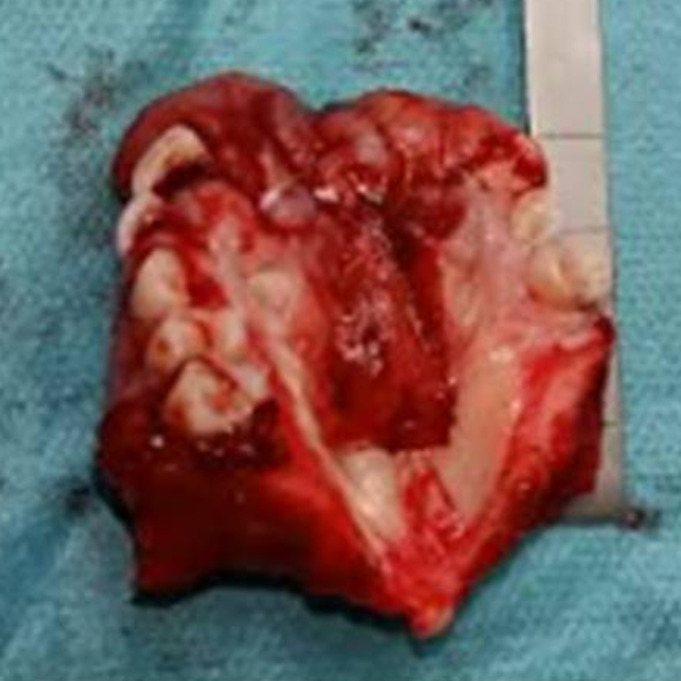
excised tumor including partial maxillectomy from tooth 15 to 25 and part of the hard palate

The patient underwent tumor resection under general anesthesia respecting 2cm security margin: partial maxillectomy from tooth 15 to 25 was done and a part of vestibule and hard palate were removed (Figure 3). Neck dissection wasn´t performed as there was no clinical or radiologic evidence of lymph node metastasis. Afterwards, the patient was referred for dental care, prosthetic reconstruction and adjuvant radiotherapy. Nine months following the surgery, check-up showed absence of local recurrence, lymphatic and distant metastasis.

## Discussion

Over 90% of all melanomas occur on the skin surfaces, while only 1% of melanomas arise from mucosal surfaces [1]. The most common site for mucosal melanoma is the head and neck region (55%), followed by the anal/rectal region (24%), female genital tract (18%) and urinary tract (3%) [1]. Less than 2% of all melanomas lack pigmentation. However, in the oral mucosa, up to two-thirds of cases are amelanotic [6]. Amelanotic malignant melanoma (AMM) was defined as a tumor composed of non-pigmented melanocytes; however, in other reports, a tumor that lacks pigmentation clinically and has melanin pigmentation histopathologically is also included in this category [7].

When pigmentation is absent, there is usually nothing else to trigger the clinician´s suspicion of malignant melanoma [7]. Also, the surface of the lesion may be smooth which can mislead the clini-cian to diagnose it as a benign tumor [8]. Symptoms of oral melanomas vary and include a bleeding lump and rarely, pain. Unfortunately, these symptoms present relatively late in the course of the disease, and by this time, significant vertical invasion of the tumor cells into the underlying tissues has already occurred [9]. As in our study, the patient had consulted after three months, only when the mass became painful.

As AMM can clinically mimic any other benign or malignant lesion, a biopsy is mandatory to establish the diagnosis. Histologically, it also may simulate a variety of other neoplasms, called by some authors as the “great pretender”. Histological differential diagnoses include spindle cell malignancies of mesenchymal tissue, lymphoma, amela¬notic melanoma and undifferentiated carcinoma. In these cases, immune-histochemical techniques provide a valuable tool for securing the correct diagnosis [5]. The majority of AMM is S100 and vimentin positive, even if it is not pathognomonic. Melanocyte-specific markers, including HMB-45, melan-A or anti-tyrosinase, are positive with variable incidence and intensity [4]. In the present case, only immunohistochemistry could assess the diagnosis of oral malignant melanoma.

The treatment of primary gingival melanoma is controversial. Surgery with complete excision and adequate negative margins seems to be the preferred treatment for localized oral melanoma. Therapeutic neck dissection is indicated for lymph node metastasis in the neck. However, elective lymph node dissection in local disease is not recommended. Recently, surgical excision with a course of IL-2 as adjunctive therapy to prevent or limit recurrence has been proposed. Although melanoma is not highly radiosensitive, patients have occasionally exhibited a good response to radiation therapy, particularly in early melanomas. It reduces the rate of local recurrence without impact on overall survival [10].

No particular type of treatment appears to improve the survival [4]. It was stated that amelanotic melanomas had a 20% survival at 3 years, whereas pigmented melanoma had a 58% survival at 3 years: a significant difference [6]. These results show that if the prognosis of oral melanoma is poor, its amelanotic variant has even poorer prognosis because it exhibits a more aggressive biology coupled to a delay in establishing the correct diagnosis. Also, the clinical aggressiveness of AMM is associated with a propensity for rapid growth and a high incidence of local recurrence and metastasis [6]. The prognosis may improve with early diagnosis by immune-histochemical examination and wide local excision [5].

## Conclusion

In our case, lack of pigmentation made the diagnosis of oral malignant melanoma difficult and immunohistochemistry examination was required to establish the proper diagnosis. Although oral localization of malignant melanoma is rare, especially its amelanotic variant, but it must be kept in mind because it´s an extremely aggressive and deadly tumor. Early detection by histological and immunochemical examination combined to wide resection are the keys to improving the survival for patients with oral amelanotic melanoma.
